# A Comprehensive Ecotoxicity Study of Molybdenum Disulfide Nanosheets versus Bulk form in Soil Organisms

**DOI:** 10.3390/nano13243163

**Published:** 2023-12-18

**Authors:** Joana Santos, Angela Barreto, Cristiana Fernandes, Ana Rita R. Silva, Diogo N. Cardoso, Edgar Pinto, Ana L. Daniel-da-Silva, Vera L. Maria

**Affiliations:** 1 Department of Biology & Centre for Environmental and Marine Studies (CESAM), University of Aveiro, 3810-193 Aveiro, Portugal; 2 Department of Environmental Health, ESS, Polytechnic of Porto, Rua Dr. António Bernardino de Almeida, 400, 4200-072 Porto, Portugal; 3 LAQV/REQUIMTE, Department of Chemical Sciences, Faculty of Pharmacy, University of Porto (FFUP), Rua de Jorge Viterbo Ferreira n° 228, 4050-313 Porto, Portugal; 4 Department of Chemistry & Aveiro Institute of Materials (CICECO), University of Aveiro, 3810-193 Aveiro, Portugal

**Keywords:** nanotoxicity, engineered nanomaterials, multi-endpoint approach, terrestrial invertebrates, comet assay, genotoxicity

## Abstract

The increasing use of molybdenum disulfide (MoS_2_) nanoparticles (NPs) raises concerns regarding their accumulation in soil ecosystems, with limited studies on their impact on soil organisms. Study aim: To unravel the effects of MoS_2_ nanosheets (two-dimensional (2D) MoS_2_ NPs) and bulk MoS_2_ (156, 313, 625, 1250, 2500 mg/kg) on *Enchytraeus crypticus* and *Folsomia candida*. The organisms’ survival and avoidance behavior remained unaffected by both forms, while reproduction and DNA integrity were impacted. For *E. crypticus*, the individual endpoint reproduction was more sensitive, increasing at lower concentrations of bulk MoS_2_ and decreasing at higher ones and at 625 mg/kg of 2D MoS_2_ NPs. For *F. candida*, the molecular endpoint DNA integrity was more impacted: 2500 mg/kg of bulk MoS_2_ induced DNA damage after 2 days, with all concentrations inducing damage by day 7. 2D MoS_2_ NPs induced DNA damage at 156 and 2500 mg/kg after 2 days, and at 1250 and 2500 mg/kg after 7 days. Despite affecting the same endpoints, bulk MoS_2_ induced more effects than 2D MoS_2_ NPs. Indeed, 2D MoS_2_ NPs only inhibited *E. crypticus* reproduction at 625 mg/kg and induced fewer (*F. candida*) or no effects (*E. crypticus*) on DNA integrity. This study highlights the different responses of terrestrial organisms to 2D MoS_2_ NPs versus bulk MoS_2_, reinforcing the importance of risk assessment when considering both forms.

## 1. Introduction

Nanotechnology is a cutting-edge scientific field, which deals with materials at the nanometer scale, offering a wide range of applications [1]. Among these, molybdenum disulfide (MoS_2_) nanomaterials are—in general—two-dimensional (2D) transition metal dichalcogenides, which have been applied in various fields, such as electronics and optoelectronics, energy, biomedicine and environmental applications (e.g., as absorbents and catalysts) [2,3,4]. Nowadays, MoS_2_ is a widely used solid lubricant, and it can be released in the form of nanoparticles (NPs) from readily available commercial products, such as brake grease, lubricating agents and hydraulic fluids [5]. The intensive production and widespread application have inevitably resulted in the release of MoS_2_ NPs into terrestrial and aquatic ecosystems, thereby exposing organisms to this material within the environment [6].

However, the understanding of the impact of MoS_2_ NPs on organisms remains limited. MoS_2_ NPs have been shown to affect the survival (LC_50_ (50% lethal concentration) = 50 µg/mL) of weaver ants *Oecophylla smaragdina*, and they induced oxidative-stress-mediated apoptosis as well as behavioral alterations in foragers fed with MoS_2_ NPs [5]. Furthermore, MoS_2_ NPs have been found to alter the intestinal metabolic profiles by changing the microbial community and inducing direct intestine toxicity in mice after oral exposure [7]. However, when applied to rice plants *Oryza sativa* L., at a dose of 100 mg/kg, MoS_2_ NPs exerted no toxicity while enhancing the group of plant-growth-promoting bacteria in soil [8]. Despite some ecotoxicological investigations into the effects of MoS_2_ NPs, the material’s potential acute and chronic hazards remain incompletely understood, especially concerning terrestrial animals.

Soils and sediments are recognized to be the final and main reservoirs of NPs [9]; however, to our knowledge, there is currently a gap in research reporting the (predicted) environmental concentrations of MoS_2_ NPs or addressing the potential toxic effects of MoS_2_ NPs on soil organisms, such as *Enchytraeus crypticus* and *Folsomia candida*. *E. crypticus* is a widely distributed terrestrial species, vital for the functioning of soil ecosystems and a standard model in soil ecotoxicology [10]. Previous studies have successfully applied *E. crypticus* for the risk assessment of various types of NPs, including boron and vanadium [11,12], silver [10,13] and nickel [14]. *F. candida*—another abundant and widespread soil species—plays a role in decomposition and mineralization processes and is commonly used as a model organism in terrestrial ecotoxicological studies [15]. Moreover, *F. candida* has been employed in the study of toxic effects of NPs of different materials, such as zinc and copper [15], gold [16] and copper oxide [17].

This work aims to unravel the impact of 2D MoS_2_ NPs exposure on soil organisms—*E. crypticus* and *F. candida*—by assessing their survival, reproduction and avoidance behavior. Additionally, the comet assay, which is a genotoxicity tool, is employed to gain a deeper understanding of MoS_2_ NPs toxicity. It is well documented that engineered NPs exhibit significant alterations in their physicochemical, mechanical and biological characteristics in comparison to their corresponding bulk forms, potentially inducing more toxicity [1]. Therefore, this study includes an assessment of the relative toxicity of MoS_2_ in the form of 2D MoS_2_ NPs (nanosheets) and in the form of micrometric material (bulk), evaluating the sensitivities of the species (*E. crypticus* versus *F. candida*) to both materials.

## 2. Materials and Methods

### 2.1. Test Species

Laboratory *E. crypticus* (Oligochaeta) and *F. candida* (Collembola) cultures were used for the exposure tests. More information in relation to the cultures’ maintenance can be found in Supplementary Information. Adult *E. crypticus* with visible clitellum and similar sizes were selected for all exposure tests performed in this study. Age-synchronized *F. candida* (juveniles, age 10–12 days) were selected for the reproduction tests, while adults with similar sizes were selected for the avoidance and comet assays.

### 2.2. Test Medium

Natural standard LUFA 2.2 soil (Speyer, Germany) was used for all the tests performed. The main characteristics of LUFA 2.2 soil are described in Supplementary Information. Soil was dried (48 h; 60 °C) before use.

### 2.3. Test Materials—Characterization and Quantification

Commercial MoS_2_ NPs were acquired from Sigma-Aldrich (CAS number: 1317-33-5; Product number: 804169; St. Louis, MO, USA), with the average particle size—as specified by the supplier—of 90 nm and a purity of 99 wt. %. Bulk MoS_2_ material was also acquired from Sigma-Aldrich (CAS number: 1317-33-5; Product number: 234842; St. Louis, MO, USA) and was specified by the supplier to have a particle size < 2 μm and a purity of 98 wt. %.

The morphology and size of MoS_2_ NPs were examined using scanning and transmission electron microscopy (SEM and TEM, respectively), employing a STEM HD2700 electron microscope (Hitachi, Tokyo, Japan) operating at 300 kV. SEM micrographs of MoS_2_ bulk material were obtained using a Hitachi SU-70 instrument (Hitachi, Tokyo, Japan) operating at 15 kV. Samples for the STEM microscope were prepared by evaporating the diluted suspensions of MoS_2_ NPs onto a copper grid coated with an amorphous carbon film. For SEM analysis, bulk MoS_2_ samples were prepared by placing a small volume of suspension diluted in ethanol onto a glass slide glued to the sample holder using double-sided carbon tape, followed by carbon sputter coating. Particle size measurement was performed using ImageJ software version 1.46. The average size of MoS_2_ materials was determined by considering the largest dimension of the particles.

Quantification of bulk and MoS_2_ NPs in the experimental media was performed using inductively coupled plasma mass spectrometry (ICP-MS). Soil samples were subjected to microwave-assisted acid digestion, following the EPA method 3051A [18]. Sample solutions were analyzed using iCAP™ Q (Thermo Fisher Scientific, Bremen, Germany) ICP-MS equipment. The elemental isotopes (m/z ratios) ^95^Mo and ^98^Mo were measured for analytical determinations, and ^71^Ga and ^103^Rh were monitored as internal standards. For analytical quality control, the certified reference material ISE 918 (sandy soil, supplied by WEPAL) was subjected to the same procedure as the samples. The obtained results were in very good agreement (recovery: 101 ± 5.7%) with the reported values.

### 2.4. Soil Spiking

The control soil (0 mg/kg of MoS_2_) was prepared by adding deionized water to adjust to the adequate moisture content (50% of the soil maximum water-holding capacity (WHC)). To achieve the final tested concentrations in soil, MoS_2_ NPs or bulk powder were weighted replicate by replicate, incorporated in the dry soil and homogeneously mixed in each replicate. Soil moisture was subsequently adjusted to 50% of the WHC.

### 2.5. Reproduction Tests

The nominal concentrations selected for the reproduction tests were 0, 156, 313, 625, 1250 and 2500 mg of MoS_2_ NPs or bulk/kg soil dry weight (DW). For the enchytraeid reproduction test, the standard OECD guidelines [19] were followed. For the collembolan reproduction test, the standard OECD guidelines [20] were followed. More details concerning the reproduction tests can be found in Supplementary Information. For both species, the tests ran at 20 ± 1 °C and a 16 h light: 8 h dark photoperiod. Food and water were replenished every week. Four replicates (n = 4) per experimental condition were used. For enchytraeids [19] and collembolans [20], the time exposure length was 21 and 28 days, respectively.

### 2.6. Avoidance Tests

Based on the results from the reproduction tests, the nominal concentrations selected for the avoidance tests were 0, 156, 625, 1250 and 2500 mg of MoS_2_ NPs or bulk/kg soil DW. The avoidance assays consisted of 2 days of exposure at 20 ± 1 °C and a photoperiod of 16 h light: 8 h dark. Five replicates (n = 5) were applied per experimental condition. For *E. crypticus*, the avoidance test was performed following the earthworm avoidance test guidelines [21]. For *F. candida*, the avoidance test guidelines ISO 17512-2 [22] were followed. More details pertaining to the avoidance tests can be found in Supplementary Information. The percentage of avoidance (A) per experimental condition was calculated as A = (C − S)/N × 100, where C is the number of organisms in control soil; S represents the number of organisms in spiked soil; and N is the total number of organisms used per replicate (10 for *E. crypticus*; 20 for *F. candida*).

### 2.7. Comet Assay Technique

The exposure conditions for the genotoxicity assessment assays were similar for both species studied. Based on the results from the reproduction tests, the nominal concentrations selected for the comet assays were 0, 156, 1250 and 2500 mg of MoS_2_ NPs or bulk/kg soil DW. Briefly, 25 adult organisms per replicate were introduced into test vessels containing 20 g of moist soil and food supply (25 mg of autoclaved oats for *E. crypticus* and 11 mg of dried baker’s yeast for *F. candida*). The test conditions were 20 ± 1 °C and a 16 h light: 8 h dark photoperiod. Five replicates per experimental condition/sampling time were used (n = 5), and sampling was performed on days 2 and 7. For both species, the comet assay technique was performed under yellow light conditions to prevent additional DNA damage.

The comet assay technique with *E. crypticus* was performed according to the optimization by Maria et al. (2018) [23]. The organisms were collected from the soil, rinsed in ISO water, maintained for 30 min and transferred to cold phosphate-buffered saline (PBS, on ice). Afterward, the organisms’ pools were chopped with scissors (15 times) to obtain a cell suspension. Cold cell suspension (40 µL) was mixed with 140 µL of 1% low melting point (LMP) agarose at 37 °C. This mixture was immediately placed on a glass microscope slide pre-coated with 1% normal melting point (NMP) agarose. A glass coverslip was then gently placed on top. The slides were maintained on ice for a minimum of 10 min. In vitro positive controls were performed, adding 25 μL of H_2_O_2_ (75 μM) to the gel containing a cellular suspension obtained from the control group, covered with a coverslip, kept on ice and exposed for 15 min at 4 °C. All slides without the coverslips were immersed in a lysis solution (overnight). The next day, slides were placed in a tank with electrophoresis buffer for alkaline treatment (20 min); the electrophoresis step was performed using the same buffer for 15 min at a voltage of 25 V and a current of 300 mA. At the end of electrophoresis, slides were neutralized (using cold PBS) and washed (using cold distilled water).

The comet assay technique with *F. candida* was conducted following the procedure described by Cardoso et al. (2017) [24]. For each replicate, soil was relocated to a glass vessel, using distilled water to fill the vessel; then, the collembolans were gently moved to a container similar to the culture boxes with plaster of Paris and charcoal. Afterward, collembolans were transferred using a suction device to a Petri dish over an ice bed to minimize organisms’ mobility. After a 10 min interval on ice, individual collembolan heads were detached from their bodies using a surgical scalpel. Subsequently, both the heads and the remaining bodies of all 25 organisms were added to a solution of PBS containing 10% dimethyl sulfoxide (DMSO) and 20 mM of ethylenediaminetetraacetic acid (EDTA). Before centrifuging the samples at 200 RCF for 20 min at 4 °C, organisms were mechanically disintegrated for a few seconds. Then, the supernatant was removed, leaving only the pellet. Following this, the pellet was resuspended by gently tapping the microtube. Subsequently, 20 µL from each sample was mixed with 140 µL of 1% LMP at 37 °C. The two components (sample and LMP agarose) were added onto microscope glass slides previously coated with 1% NMP agarose. A coverslip was then gently placed on top. The slides were maintained on ice for a minimum of 10 min. Simultaneously, additional slides were prepared as positive controls by adding PBS solution with H_2_O_2_ (200 μM) to each slide. These positive control slides were also kept on ice for 15 min to ensure that DNA damage had occurred. Afterward, the coverslips were removed, and the glass slides were immersed in a lysis solution at 4 °C for at least 1 h. Subsequently, the slides were transferred to the electrophoresis equipment, previously filled with electrophoresis solution. Electrophoresis was conducted at an electric current of 250 mA (25 V) for 20 min. Following electrophoresis, the slides were neutralized in three sequential washes with 0.4 M Tris-HCl. Finally, the slides were briefly submerged in absolute ethanol for 10 s.

After 1-day air-drying in a dark environment, the slides with gels (prepared from *E. crypticus* and *F. candida*) were stained with DAPI (4′,6-diamidino-2-phenylindole) for further visualization. Lastly, the slides were observed to score 100 nucleoids/replicate. *E. crypticus* and *F. candida* DNA damage was classified into five comet classes, according to the tail intensity and length, from 0 (no tail) to 4 (almost all DNA in the tail). A DNA damage index (DI), in arbitrary units, was assigned to each replicate, and consequently for each treatment, using the formula

DI = (0 × n0) + (1 × n1) + (2 × n2) + (3 × n3) + (4 × n4)

where n = number of cells in each class. DI can range from 0 (minimum damage) to 400 (maximum damage).

### 2.8. Data Analysis

Graphics and statistics analyses were conducted using the Sigma Plot 12.5 software. Significant differences were considered at a significance level (*p*) < 0.05. More details regarding data analysis can be found in Supplementary Information.

## 3. Results and Discussion

### 3.1. Molybdenum Disulfide Characterization and Quantification

**Figure 1:**
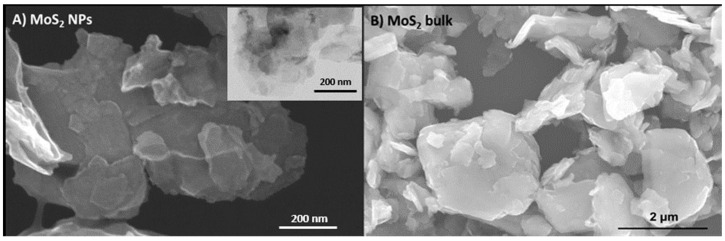
Scanning electron microscopy (SEM) and transmission electron microscopy (TEM) (inset) images of two-dimensional molybdenum disulfide (MoS_2_) nanoparticles (NPs) (**A**) and SEM image of bulk MoS_2_ (**B**).

MoS_2_ NPs exhibited a 2D structure with significant aggregation (Figure 1A and Figure S1). The average size of 2D MoS_2_ NPs was 193.8 ± 69.6 nm. This size, assessed through electron microscopy, exceeded the manufacturer’s specification of 90 nm due to the presence of extended 2D structures, resulting in larger particle sizes. Similarly, bulk MoS_2_ material displayed highly aggregated lamellar structures (Figure 1B and Figure S1). The measured average length for bulk MoS_2_ was 1.76 ± 0.57 µm, which was in accordance with the size specified by the manufacturer (<2 μm).

At 0 h, the measured MoS_2_ concentrations were in agreement with the nominal concentrations for both NPs and bulk forms (Table 1).

### 3.2. Adult Survival and Reproduction

**Figure 2:**
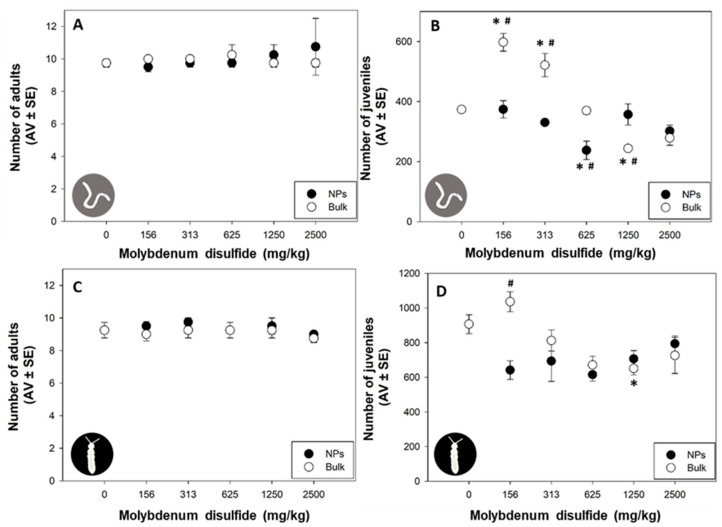
Effects on survival (number of adults) and reproduction (number of juveniles) of *Enchytraeus crypticus* (**A**,**B**) after 21 days and *Folsomia candida* (**C**,**D**) after 28 days of exposure to two-dimensional molybdenum disulfide (MoS_2_) nanoparticles (NPs) and bulk MoS_2_ in LUFA 2.2 soil. Data are expressed as average value (AV) ± standard error (SE). * Significant differences with control group—0 mg/kg (*p* < 0.05). **#** Significant differences between the two forms (NPs versus bulk) within the same concentration (*p* < 0.05).

Research on the toxicity of engineered NPs is still in development. Specifically, there is a significant knowledge gap regarding the potential environmental impacts of MoS_2_ NPs applied in novel technologies [25]. In this study, 2D MoS_2_ NPs or bulk MoS_2_ did not significantly affect the adult survival of *E. crypticus* (*p* > 0.05; Figure 2A) or *F. candida* (*p* > 0.05; Figure 2C) across all tested concentrations. However, the reproductive outcome of both species was significantly affected by some MoS_2_ tested concentrations (*p* > 0.05; Figure 2B,D). This suggests that the reproductive outcome of these soil organisms is more vulnerable to 2D MoS_2_ NPs and bulk MoS_2_ than the survival response, as already found in other studies with NPs [26,27,28]. García-Gómez et al. (2014) also reported no effect on the survival of *Eisenia fetida* after 28 days of exposure to zinc oxide NPs and bulk forms (1000 mg/kg), but a significant effect on reproduction was observed (59 and 43% inhibition for NPs and bulk forms, respectively) [26]. Santos et al. (2017) reported that *E. crypticus* survival was not affected by nickel NPs exposure (1500 mg/kg), but the number of juveniles decreased after exposure to concentrations ≥600 mg/kg of nickel NPs [27]. For *F. candida,* similar results were observed, with no effect on survival after zinc oxide NPs exposure (6400 mg/kg) but reduced reproduction (EC_50_ (50% effect concentration) = 1695 mg/kg) [28].

To our knowledge, only three in vivo studies assessed MoS_2_ NP toxicity [5,7,29]. Affected survival (LC_50_ = 50 µg/mL), oxidative stress during cellular toxicity and behavioral alterations were found in weaver ants *Oecophylla smaragdina* feed with MoS_2_ NPs [5]. Ants feed with MoS_2_-NP also showed decreased hemocyte count, increased apoptotic activity and reactive oxygen species (ROS) levels, increased activity of superoxide dismutase (SOD) and lipid peroxidation levels, and decreased activities of catalase (CAT) and glutathione-*S*-transferase (GST) [5]. MoS_2_ NPs also enhanced biomass and β-carotene production in the microalgae *Dunaliella salina,* suggesting that MoS_2_ NPs might change the rudimentary ecological composition in the ocean [29]. MoS_2_ NPs via oral exposure in mice for 90 days (15 and 150 mg of MoS_2_ per kg food) altered the intestinal metabolic profiles by changing the microbial community and inducing direct intestine toxicity, such as mucosal hemorrhage, villus shortening and edema of the intestinal wall [7].

In our study, for *E. crypticus* exposed to bulk MoS_2_, a non-monotonic “U”-shaped response was observed, with a significant increase in reproduction for the lowest tested concentrations (156 and 313 mg/kg; *p* < 0.05; Figure 2B) and a decrease for a higher concentration (1250 mg/kg; *p* < 0.05; Figure 2B). This unique pattern suggests a complex and dose-dependent relationship between *E. crypticus* and bulk MoS_2_ exposure. The initial increase in reproduction at lower concentrations may indicate a stimulatory or adaptive response, potentially linked to hormesis. However, as the concentration rises beyond a certain threshold, a detrimental impact emerges, leading to a subsequent decrease in reproduction. Further investigation is warranted to elucidate the underlying mechanisms behind this non-monotonic response, exploring potential factors, such as hormesis, stress response or specific interactions between *E. crypticus* and bulk MoS_2_. This nuanced understanding is crucial for deciphering the intricacies of the observed reproductive dynamics and contributing to a more comprehensive comprehension of the ecological implications of bulk MoS_2_ exposure on soil organisms. 2D MoS_2_ NPs only inhibited *E. crypticus* reproduction at 625 mg/kg (*p* < 0.05; Figure 2B). The results indicated that reproduction was significantly affected by both 2D MoS_2_ NPs and bulk MoS_2_, but with a different response pattern, with bulk MoS_2_ being more toxic for both species. Indeed, for reproduction, significant differences were found between the two forms (NPs versus bulk) for 156, 313, 625 and 1250 mg/kg for *E. crypticus* and for 156 mg/kg for *F. candida* (*p* < 0.05; Figure 2A,B). García-Gomez et al. (2014) also reported a different response pattern for the fertility of *Eisenia fetida* exposed to zinc oxide NPs and bulk forms: at 1000 mg/kg, zinc oxide NPs decreased fertility by 72%, whereas bulk zinc oxide increased the offspring number per cocoon by 36% [26]. In the present study, for *F. candida*, fewer effects were detected, with significant decrease in reproduction only at 1250 mg/kg of bulk MoS_2_ (*p* < 0.05; Figure 2D) and no effect of 2D MoS_2_ NPs exposure. An effect on reproductive outcome is of utmost importance because even small changes in reproduction can seriously deplete soil organism populations over time [30]. Organisms under different levels of stress may attempt to “trade them-off” at the expense of energy for biological processes, such as reproduction [30]. Therefore, maintaining the detoxification processes of organisms in contaminated soil may compromise reproduction success [30]. Moreover, MoS_2_ nanosheets were reported to impact the energy metabolism of *Eisenia fetida*, with pyruvate metabolism and glycolysis being the most significantly impacted pathways [31]. Further studies assessing the energy metabolism and detoxification processes in *F. candida* and *E. crypticus* exposed to MoS_2_ are needed to confirm this possibility.

Soil will be the main sink for engineered NPs in terrestrial environments [8], and MoS_2_ nanomaterials may undergo oxidation and dissolution processes in soil, releasing soluble molybdenum (Mo) and sulfur (S) species [3,8]. Therefore, soil organisms will interact not only with particulate MoS_2_ but also with other Mo species, including ionic species [8]. However, the toxicity and risk of Mo to soil organisms are not well known [32]. Only a few studies including individual responses (survival and reproduction) were previously performed [32,33,34]. Mo affected soil invertebrate survival only at high concentrations: LC_50_ was >3200 mg/kg for *Eisenia andrei*, *F. candida* and *E. crypticus* [34], and less than 50% *Eisenia andrei* mortality was reported for 3200 mg/kg of Mo [32]. However, reproduction was reported to be more sensitive to Mo exposure: EC_50_ = 129–2378 mg/kg for *Eisenia andrei*, 72–3396 mg/kg for *F. candida* and 301–2820 mg/kg for *E. crypticus* [34]. Based on these results, it is possible that the 2D MoS_2_ NPs and bulk effects found in our study on *E. crypticus* and *F. candida* reproduction may be due to the Mo ionic species released from both forms instead of a particle-specific effect. Our hypothesis is that the dominant mechanism for the observed adverse effects appears to be the leaching of Mo ionic species, which seems to occur more quickly for bulk MoS_2_. However, the observed toxic effects certainly result from other mechanisms behind this. In general, smaller particles (of the same material) have a larger surface area and can therefore leach Mo ionic species more quickly, leading to a greater release of ions (faster or greater quantity). Moreover, the toxicity of metal-based NPs may result from a synergistic action of the metal ions and the particle effect, resulting in higher nanoform toxicity [35]. Therefore, more studies investigating the leaching rates of Mo ionic species in both forms are needed to better understand the different mechanisms involved in the toxicity of MoS_2_ NPs versus bulk form for the tested species.

### 3.3. Avoidance Behavior

**Figure 3:**
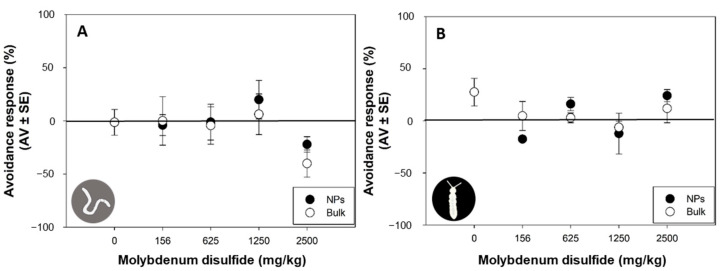
Avoidance responses (%) of *Enchytraeus crypticus* (**A**) and *Folsomia candida* (**B**) after 2 days of exposure to two-dimensional molybdenum disulfide (MoS_2_) nanoparticles (NPs) and bulk MoS_2_ in LUFA 2.2 soil. Data are expressed as average value (AV) ± standard error (SE).

Avoidance behavior must be understood as a protective mechanism, since organisms may protect themselves from further toxicity if they avoid the contaminated soil [36]. Previous studies have reported that metal-based NPs induced avoidance to earthworms: *Eisenia fetida* avoided soils contaminated with 200 and 500 mg/kg of lanthanum oxide NPs [37] and aluminum oxide NPs at concentrations higher than 5000 mg/kg [38]; *Enchytraeus albidus* exhibited stronger avoidance behavior (EC_50_ = 241 mg/kg) after exposure to copper NPs [39]. However, in this study, after 2 days of exposure, both 2D MoS_2_ NPs and bulk MoS_2_ induced no significant avoidance response in *E. crypticus* (*p* > 0.05; Figure 3A) and *F. candida* (*p* > 0.05; Figure 3B).

When organisms cannot escape contaminated environments, they can become intoxicated, which can affect other biological responses [36]. A neurotoxic effect induced by exposure to contaminated soil can result in non-response in the organisms, which are unable to avoid it, being exposed to the contaminated soil [36]. For example, inhibiting the neurotransmitter acetylcholinesterase (AChE) has been linked to the absence of avoidance behavior [12,40]. Furthermore, AChE activity inhibition could be due to NMs (physical) interaction with the enzyme structure and due to the dissolved ions from bulk particles [41]. Thus, it is possible that the observed no avoidance may have been caused by MoS_2_ inhibition of AChE, and therefore, the organisms were not able to avoid the contaminated soil due to paralysis. However, future studies evaluating *E. crypticus* and *F. candida* AChE performance are needed to validate the hypothesis, whereby inhibition of locomotion prevailed over escaping.

### 3.4. DNA Damage

**Figure 4:**
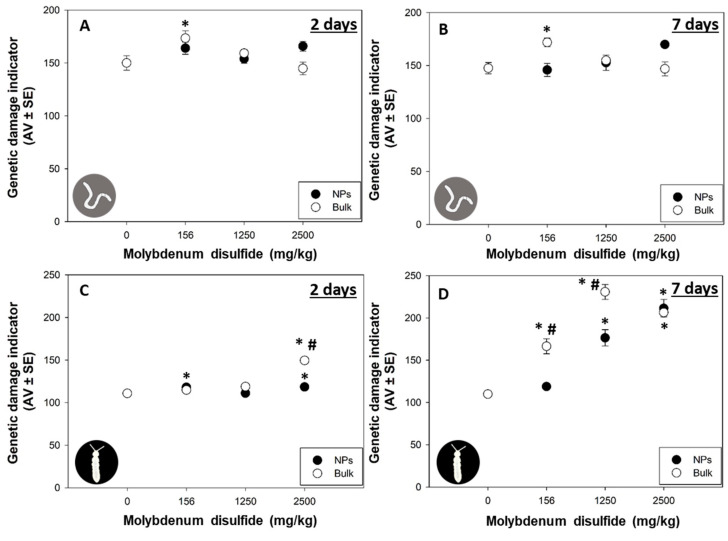
The DNA damage measured as genetic damage indicator (in arbitrary units) of *Enchytraeus crypticus* (**A**,**B**) and *Folsomia candida* (**C**,**D**) after 2 and 7 days of exposure to two-dimensional molybdenum disulfide (MoS_2_) nanoparticles (NPs) and bulk MoS_2_ in LUFA 2.2 soil. Data are expressed as average value (AV) ± standard error (SE). * Significant differences with the corresponding control group—0 mg/kg (*p* < 0.05). # Significant differences between the two forms (NPs versus bulk) within the same concentration (*p* < 0.05).

The comet scale considered can be found in Supplementary Information (Figure S2). After 2 days of exposure, in *E. crypticus*, 156 mg/kg of bulk MoS_2_ significantly induced DNA damage compared to the control (*p* < 0.05; Figure 4A), and this induction remained unaltered after 7 days of exposure (*p* < 0.05; Figure 4B). Considering 2D MoS_2_ NPs exposure, no effects on the DNA integrity of *E. crypticus* were found for the tested concentrations and for both days (*p* > 0.05; Figure 4A,B).

At 2 days of exposure, in *F. candida*, 2500 mg/kg of bulk MoS_2_ significantly induced DNA damage (*p* < 0.05; Figure 4C). However, after 7 days of exposure, all the tested concentrations of bulk MoS_2_ significantly induced DNA damage in *F. candida* (*p* < 0.05; Figure 4D). For the 2D MoS_2_ NP exposure, a significant but slight induction in the DNA damage of *F. candida* was detected for 156 and 2500 mg/kg (*p* < 0.05; Figure 4C), while after 7 days of exposure, a significant and larger induction was detected at 1250 and 2500 mg/kg (*p* < 0.05; Figure 4D). Moreover, significant differences were found between the two forms (NPs versus bulk) for 2500 mg/kg at 2 days and for 156 and 1250 mg/kg at 7 days (*p* < 0.05; Figure 4C,D).

NPs have been reported to have the ability to enter cells and interact directly with DNA through direct binding to DNA or DNA repair enzymes, which may promote DNA instability or indirectly interact with DNA by producing ROS, which can induce DNA oxidation and depletion of antioxidant defense [42]. Exfoliated MoS_2_ induced ROS-independent oxidative stress generation and depolarization of bacterial membrane (*Staphylococcus aureus* and *Pseudomonas aeruginosa*) [43], while in freshwater microalgae *Chlorella vulgaris*, the ROS levels in cells exposed to 1 mg/L of MoS_2_ nanosheets were evidently boosted [3]. Normally, NPs are more reactive than their correspondent bulk form, resulting in higher toxicity [1]. In the present study, although both 2D MoS_2_ NPs and bulk MoS_2_ induced DNA damage, the bulk MoS_2_ exhibited an earlier effect (2 days) at a lower concentration (156 mg/kg) compared to the NPs. Maria et al. (2017) also showed an earlier effect of silver nitrate salt compared with silver NPs, with the bulk form causing genotoxicity after 3 days and NPs only after 7 days [23]. He et al. (2020) showed that exposure to zinc oxide NPs resulted in lower impact on the metabolic response of *E. crypticus* than exposure to the correspondent bulk form [35]. In our study, the results of DNA integrity are in line with data from the previously mentioned studies, which report that bulk forms of various metals induced more toxic effects compared to the corresponding NPs. The NP characteristics and/or behavior in the media may play an important role in changing their toxicity when they accumulate in the organisms [35]. The 2D MoS_2_ NPs probably remained as particles and delayed their ion leaching, resulting in a later effect on *E. crypticus* and *F. candida* DNA damage. This hypothesis suggests that 2D MoS_2_ NPs genotoxicity is primarily due to ion leaching rather than the particles themselves. However, further investigations addressing the behavior and dissolution dynamics of MoS_2_ NPs within the soil matrix are required to enhance our comprehension of these findings.

### 3.5. Nanoparticle versus Bulk Effects—An Overview

NPs have unique physical properties distinct from the properties of their corresponding bulk forms due to increased relative surface area per volume unit and the dominance of quantum effects [25]. As the size decreases, the surface energy of the particles is expected to increase, which normally results in enhanced dissolution and solubility of NPs as compared to the bulk form [44]. Moreover, NPs are absorbed more highly into the respiratory, skin and gastrointestinal systems than the bulk form because of their size and surface modifications [44]. Thus, NPs interact with biological systems through different mechanisms of action and normally have different tissue distribution patterns than their bulk form, resulting in higher toxicity [44]. Indeed, numerous studies have demonstrated that, once in the environment, nanomaterials are more toxic to organisms than their corresponding bulk counterparts [9,45,46]. A previous study with MoS_2_ nanosheets showed that the NPs form induced a higher level of toxicity than bulk MoS_2_ micromaterial, resulting in oxidative stress to soil bacteria *Bacillus cereus* and *Pseudomonas aeruginosa* [45]. Similarly, iron oxide NPs were more toxic to the plant *Oenothera biennis* than the bulk form, resulting in changes in the antioxidant and physiological systems [9]. Higher genotoxic effect was also found in the plant *Taraxacum officinale* exposed to copper oxide and zinc oxide NPs compared to their corresponding bulk counterparts [46]. However, in this study, bulk MoS_2_ induced more toxic effects on *E. crypticus* and *F. candida* compared with 2D MoS_2_ NPs. Here, although both forms impacted the same endpoints (reproduction and DNA integrity), different patterns of response were observed, with bulk MoS_2_ inducing more effects than 2D MoS_2_ NPs on the selected terrestrial species. Although both species were more affected by bulk MoS_2_, for *E. crypticus*, an individual endpoint (reproduction) was more sensitive, while for *F. candida*, a molecular endpoint (DNA integrity) was more impacted. While this study provides a valuable foundation for exploring the effects of 2D MoS_2_ NPs and bulk MoS_2_ on two distinct soil species and crucial information for risk assessment, a more comprehensive investigation into the diverse physiological aspects influencing their varying response patterns requires further research at the biochemical and molecular levels. This includes the detection of ROS and indicators related to antioxidant defense system.

## Figures and Tables

**Table 1:** Determined concentrations of two-dimensional (2D) molybdenum disulfide (MoS_2_) nanoparticles (NPs) and bulk MoS_2_ in the exposure media (LUFA 2.2 soil) at the beginning of the exposure tests (day 0). Results are expressed as average value ± standard error (n = 3).

Nominal Concentrations (mg/kg)	Measured Concentrations (mg/kg)	
	2D MoS_2_ NPs	Bulk MoS_2_
0	0.25 ± 0.01	0.25 ± 0.01
156	156 ± 3.4	149 ± 3.4
313	315 ± 5.6	306 ± 4.3
625	634 ± 20	591 ± 6.2
1250	1247 ± 31	1294 ± 34
2500	2505 ± 32	2497 ± 72

## Data Availability

Data are contained within the article and supplementary materials.

## Supplementary Materials

The following supporting information can be downloaded at: https://www.mdpi.com/article/10.3390/nano13243163/s1, Figure S1. Scanning electron microscopy (SEM) of MoS_2_ nanosheets (NPs) in a holey C-Supported Cu Grid (left side) and SEM image of bulk MoS_2_ (right side) at identical magnification. Figure S2. Comet scale. A five-class classification based on the tail DNA intensity and length, from 0 (no tail, undamaged) to 4 (almost all DNA in the tail, maximum damage).
