# Supplementary material for: A Comprehensive Ecotoxicity Study of Molybdenum Disulfide Nanosheets versus Bulk form in Soil Organisms
Source: Nanomaterials (Basel). 2023 Dec 18;13(24):3163. doi: 10.3390/nano13243163 (PMC10745638; doi:10.3390/nano13243163)
Supplement: Supplementary file 1 [file nanomaterials-13-03163-s001.zip › nanomaterials-2745956-supplementary.pdf]

A Comprehensive Ecotoxicity Study of Molybdenum Disulfide Nanosheets versus Bulk form in Soil Organisms

Joana Santos [1], Angela Barreto [1,*], Cristiana Fernandes [1], Ana Rita R. Silva [1], Diogo N. Cardoso [1], Edgar Pinto [2,3], Ana L. Daniel-da-Silva [4] and Vera L. Maria [1,*]

1 Department of Biology & Centre for Environmental and Marine Studies (CESAM), University of Aveiro, 3810-193 Aveiro, Portugal
2 Department of Environmental Health, School of Health of Polytechnic of Porto (ESS P), Rua Dr. António Bernardino de Almeida, 400, 4200-072 Porto, Portugal
3 Associated Laboratory for Green Chemistry (LAQV) of the Network of Chemistry and Technology (REQUIMTE), Department of Chemical Sciences, Faculty of Pharmacy, University of Porto (FFUP), Rua de Jorge Viterbo Ferreira n° 228, 4050-313 Porto, Portugal
4 Department of Chemistry & Aveiro Institute of Materials (CICECO), University of Aveiro, 3810-193 Aveiro, Portugal
* Correspondence: abarreto@ua.pt (A.B.); vmaria@ua.pt (V.L.M.)

Supporting Information

2. Material and Methods

2.1. Test species

Laboratorial *E. crypticus* (Oligochaeta) cultures were kept in agar plates, consisting of Bacti-Agar medium (Oxoid; Agar No. 1) and a sterilized mixture of 4 different salt solutions at final concentrations of 2 mM $CaCl_2 \bullet 2H_2O$, 1 mM $MgSO_4$, 0.08 mM KCl, and 0.75 mM $NaHCO_3$. Organisms were fed *ad libitum* with ground autoclaved oatmeal twice per week and maintained at 20 ± 1 °C with a 16 hours (h): 8 h (light: dark) photoperiod. Adults with visible clitellum and similar sizes were selected for all the exposure tests performed in this study.

F. candida (Collembola) cultures were maintained in laboratory, on a moist substrate of plaster of Paris and activated charcoal (8:1 ratio), at 20 ± 1 °C, under a photoperiod of 16 h: 8 h (light: dark). The organisms were fed once per week with dried baker's yeast (*Saccharomyces cerevisae*). Organisms age synchronized (juveniles with 10–12 days) were selected for the reproduction tests, while adults with similar sizes were selected for the avoidance and comet assays.

2.2. Test medium

The natural standard LUFA 2.2 soil (Speyer, Germany) was used for all the performed tests and had the following main characteristics, according to the supplier: pH (0.01 M $CaCl_2$) = 5.8; organic carbon = 1.71%; cation exchange capacity = 9.2 meq/100 g; maximum water-holding capacity (WHC) = 44.1%; and grain size distribution of 7.2% clay, 8% silt, and 77.5% sand. The soil was dried (48 h; 60 °C) before use.

2.5. Reproduction tests

For the enchytraeid reproduction test the standard OECD guidelines [1] were followed. The nominal concentrations selected for the reproduction tests were 0, 156, 313, 625, 1250 and 2500 mg MoS_2 NPs or bulk/kg soil dry weight (DW). Briefly, 10 adult organisms were introduced in each test container (replicate) with 20 g of moist soil and food supply (25 mg of autoclaved oats). At the test end, replicates were fixated with 96% ethanol and Bengal rose (solution at 1% in ethanol). Samples were sieved through three meshes (0.6, 0.2, and 0.1 mm) to separate individuals

from most of the soil and facilitate counting using a stereo microscope. Adult survival (number of adults) and reproduction (number of juveniles) were evaluated.

For the collembolan reproduction test, the standard OECD guidelines [2] were followed. In short, 10 individuals were placed in each test vessel (replicate), containing 30 g of moist soil and food (11 mg of dried baker's yeast). At the test end, each test vessel was flooded with water, the content was transferred to a crystallizer dish, and the surface was photographed for further counting of the organisms applying the software ImageJ version 1.46. Adult survival (number of adults) and reproduction (number of juveniles) were assessed.

For both species, tests ran at 20 ± 1 °C and a 16 h light: 8 h dark photoperiod. Food and water were replenished every week. Four replicates (n = 4) per experimental condition were used. For enchytraeids [1] and collembolans [2], the time exposure length was 21 and 28 days, respectively.

2.6. Avoidance tests

Based on the results from the reproduction tests, the nominal concentrations selected for the avoidance tests were 0, 156, 625, 1250 and 2500 mg MoS_2 NPs or bulk/kg soil DW. Avoidance assays consisted of 2 days of exposure at 20 ± 1 °C and a photoperiod of 16 h light: 8 h dark. Five biological replicates (n=5) with pools of organisms were applied per experimental condition. For *E. crypticus*, the avoidance test was performed following the earthworm avoidance test guidelines [3]. In brief, half of each container was filled with 25 g of control soil and the other half with 25 g of spiked soil. Ten adult organisms were used per replicate and placed on the separating line of each test vessel. At the end of the test, each side of the container was independently searched for worms. For *F. candida*, the avoidance test guidelines ISO 17512-2 [4] were followed. Half of each container was filled with 30 g of the control soil and the other half with 30 g of spiked soil. Twenty adult organisms were used per replicate and placed on the separating line of each test vessel. After 2 days, the soil from each half of the container was separated and placed into new vessels, flooded with water and the number of floating individuals was counted directly.

As a test validation, a dual control test was performed with both compartments of the container filled with control soil. Percentage of avoidance (A) per experimental condition was calculated as $A = (C - S) / N \times 100$, where C is the number of organisms in control soil, S represents the number of organisms on spiked soil and N is the total number of organisms used per replicate (10 for *E. crypticus*; 20 for *F. candida*).

2.8. Data analysis

Graphics and statistics analysis were made using the Sigma Plot 12.5 software. Data were tested for normality and homogeneity of variance using the Shapiro–Wilk and the Levene tests, respectively. One-way analysis of variance (ANOVA) followed by Dunnett's post hoc test was used to compare the treatments (NPs or bulk) with the control group (0 mg/kg). When data failed the normality and homoscedasticity tests, a non-parametric Kruskal–Wallis' test was performed. Two-way analysis of variance (ANOVA) was performed to compare the two forms NPs *versus* bulk, considering two independent factors: concentration and form type. Significant differences were considered for a significance level (p) < 0.05.

3. Results and Discussion

3.1. Molybdenum disulfide characterization and quantification

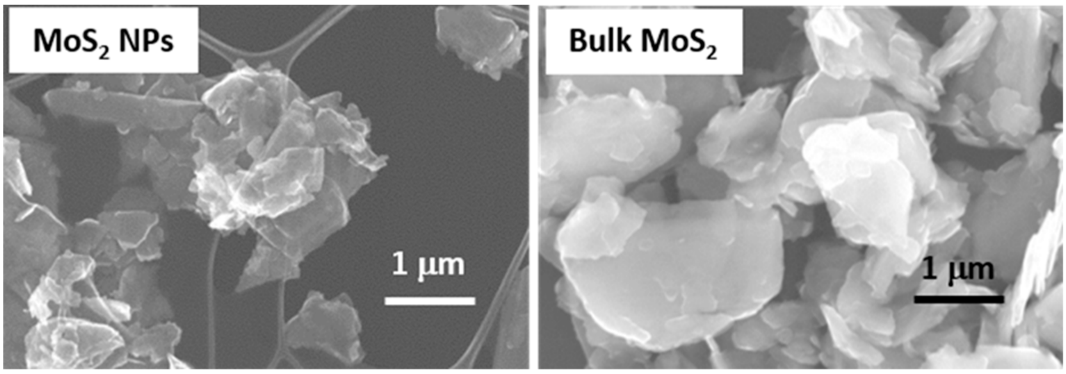

Figure S1. Scanning electron microscopy (SEM) of MoS_2 nanosheets (NPs) in a holey C-Supported Cu Grid (left side) and SEM image of bulk MoS_2 (right side) at identical magnification.

3.4. DNA damage

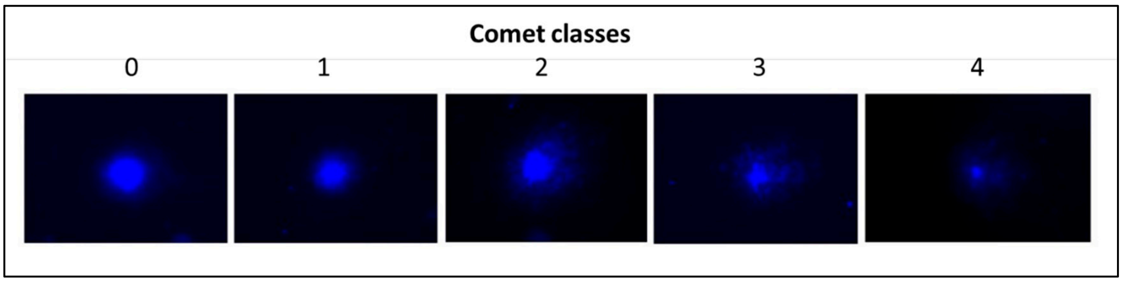

Figure S2. Comet scale. A five-class classification based on the tail DNA intensity and length, from 0 (no tail, undamaged) to 4 (almost all DNA in the tail, maximum damage).

References

1. *OECD Test 220*; Enchytraeid Reproduction Test. OECD Guidelines for the Testing of Chemicals, Section 2. OECD Publication: Paris, France, 2016; pp. 1–24.
2. *OECD Test 232*; Collembolan Reproduction Test in Soil. OECD Guidelines for the Testing of Chemicals. OECD Publication: Paris, France, 2016; pp. 1–22.
3. *ISO 17512-1:2008*; ISO Soil Quality—Avoidance Test for Determining the Quality of Soils and Effects of Chemicals on Behaviour—Part 1: Test with Earthworms (*Eisenia Fetida* and *Eisenia Andrei*). International Organization for Standardization (ISO): Geneva, Switzerland, 2008; pp. 1–25.
4. *ISO 17512-2:2011*; Soil Quality—Avoidance Test for Testing the Quality of Soils and Effects of Chemicals—Part 2: Test with Collembolans (*Folsomia candida*). Guideline. International Organization for Standardization (ISO): Geneva, Switzerland, 2011; pp. 1–13.
